# The NineTeen Complex (NTC) and NTC-associated proteins as targets for spliceosomal ATPase action during pre-mRNA splicing 

**DOI:** 10.1080/15476286.2015.1008926

**Published:** 2015-02-05

**Authors:** Rogerio Alves de Almeida, Raymond T O’Keefe

**Affiliations:** Faculty of Life Sciences; The University of Manchester; Manchester, UK

**Keywords:** ATPase, Brr2, Cwc2, NineTeen Complex, PremRNA splicing, Prp19, Prp2, Prp16, Prp43, RNA helicase

## Abstract

Pre-mRNA splicing is an essential step in gene expression that removes intron sequences efficiently and accurately to produce a mature mRNA for translation. It is the large and dynamic RNA-protein complex called the spliceosome that catalyzes intron removal. To carry out splicing the spliceosome not only needs to assemble correctly with the pre-mRNA but the spliceosome requires extensive remodelling of its RNA and protein components to execute the 2 steps of intron removal. Spliceosome remodelling is achieved through the action of ATPases that target both RNA and proteins to produce spliceosome conformations competent for each step of spliceosome activation, catalysis and disassembly. An increasing amount of research has pointed to the spliceosome associated NineTeen Complex (NTC) of proteins as targets for the action of a number of the spliceosomal ATPases during spliceosome remodelling. In this point-of-view article we present the latest findings on the changes in the NTC that occur following ATPase action that are required for spliceosome activation, catalysis and disassembly. We proposed that the NTC is one of the main targets of ATPase action during spliceosome remodelling required for pre-mRNA splicing.

## Introduction

**Figure 1.**
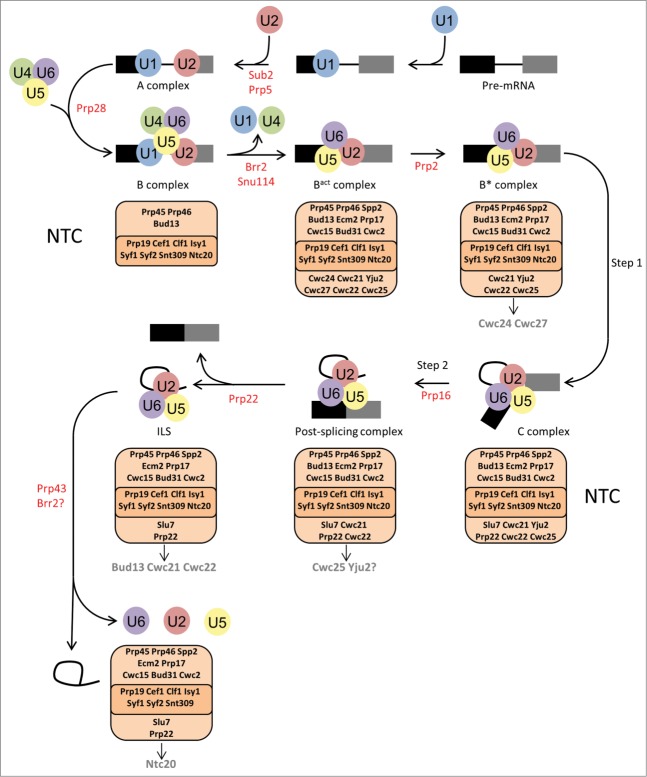
NTC protein remodelling by ATPases during the spliceosome assembly, activation and disassembly process. The pathway of complexes formed on the pre-mRNA during spliceosome assembly, activation and disassembly are indicated with arrows and the names of each complex are given below the complex. The ATPases are shown in red under the arrow for the step that each ATPase promotes. The NTC core complex (dark orange) and NTC-associated proteins (light orange) are shown below each complex that they associate with. Arrows from the NTC complex are used to indicate the proteins that leave following ATPase action. Question marks are used to indicate that experimental evidence for Yju2 removal from the NTC and Brr2 action during spliceosome disassembly is not in agreement.

Splicing of pre-mRNA (pre-mRNA) is a complex mechanism where introns are removed, and exons are joined together to form a mature mRNA competent for translation. Pre-mRNA splicing is tightly regulated and its failure is linked to various tumors, pathologies of the endocrine system and neurodegenerative disorders.^1^ This intrinsic process of splicing is mediated by a multimegadalton ribonucleoprotein complex called the spliceosome. The spliceosome consists of 5 small nuclear RNAs (snRNAs), U1, U2, U4, U5 and U6, which are associated with proteins forming ribonucleoprotein particles (snRNPs).^2,3^ The snRNPs assemble with the pre-mRNA and are then remodelled into a number of specific complexes required for the 2 steps of splicing (**Fig. 1**). The process of splicing begins when the U1 and U2 snRNAs of the U1 and U2 snRNPs recognize, by base-pairing, the 5' splice site and the branch site of the pre-mRNA, respectively, to form the A complex. Then a preformed tri-snRNP, containing U4/U6 and U5 snRNPs, joins complex A to form complex B. At the same time a protein complex associated with Prp19, named the NineTeen Complex (NTC), also joins the spliceosome.^4-6^ Next, the spliceosome goes through dramatic rearrangements to form the B^act^ then B* complexes which involves dissociation of the U1 and U4 snRNPs as well as the removal and addition of certain proteins.^7^ The B* complex is now the catalytically activated spliceosome and is competent to carry out the first step of splicing, which is attack of the 5′ splice site phosphate by the 2′ hydroxyl of the branch site adenosine. Following the first step of splicing the B* complex is then rearranged to form the C complex through the removal of proteins and the addition of proteins that promote the second step of splicing.^8^ The C complex carries out the second step of splicing which is attack of the 3′ splice site phosphate by the 3′ hydroxyl of the 5′ exon to remove the intron and join the 2 exons. The resulting splicing complex is called the post-splicing complex and this complex must be disassembled to release the mRNA, leaving the intron associated with the snRNPs as the intron-lariat spliceosome (ILS). Finally, the ILS is disassembled allowing recycling of the snRNPs for subsequent rounds of splicing. The rearrangements and conformational changes required for spliceosome assembly, activation and disassembly are catalyzed by the spliceosomal ATPases.^9^ In this “Point of View” we will describe an increasing amount of experimental evidence that identifies the NTC, and NTC-associated proteins, as targets for a number of the spliceosomal ATPases required for remodelling the spliceosome during pre-mRNA splicing. We will concentrate on the yeast *Saccharomyces cerevisiae* system where there is the most evidence to date for this idea.

There are 8 ATPases involved in splicing (**Table 1**) and they share 8 conserved motifs divided into 2 domains, RecA1 and RecA2.^9^ The spliceosomal ATPases are used to modulate RNA-RNA, RNA-protein and protein-protein interactions during the splicing cycle.^10^ The ATPases promote correct conformations of the spliceosome for progression through the 2 steps of intron removal with accuracy and fidelity. It is the second role of some of the ATPases, that of proofreading certain steps of splicing, which provides the fidelity in the splicing process.^11-15^ Spliceosome conformations are monitored by the ATPases and can be rejected if a certain complex is not formed correctly or in a timely manner. The order of action of the ATPases during splicing is Sub2, Prp5, Prp28, Brr2, Prp2, Prp16, Prp22, Prp43 and Brr2 (**Fig. 1**).^14^ Of these ATPases, the action of Prp2, Prp16, Prp22, Prp43 and Brr2 appear to be related to changes in the interactions of the NTC and NTC-associated proteins with the spliceosome. The modulation of the NTC, therefore, is essential for the progression of the spliceosome through the splicing cycle.

**Table 1.** Yeast Spliceosomal ATPases

ATPase	Family	Function
Sub2/UAP56	DEAD-box	Association of U2 snRNA with pre-mRNA
Prp5	DEAD-box	Proofreads U2-branchsite interaction
Prp28	DEAD-box	Release of U1 by disrupting the base-pairing between U1 and 5′splice site.
Prp2	DEAH-box	Release of SF3a/SF3b
Prp16	DEAH-box	Release of Yju2 and Cwc25
		U2/U6 helix I remodelling
Prp22	DEAH-box	Release of mature mRNA
Prp43	DEAH-box	Disassembly of the ILS
Brr2	Ski2-like	Disrupt U4/U6 base-pairing
		Disrupt U2/U6 base-pairing

The NTC and NTC-associated proteins are found within the B, B^act^, B*, C, post-splicing complex and ILS (**Fig. 1**).^8,16^^,^^17^ The core NTC complex nucleated by Prp19, is found in all these complexes, it is composed of 8 proteins, but an additional 18 NTC-associated proteins interact with, or co-purify with, these core proteins and can be found in one or more of the splicing complexes.^5^ At the core of the NTC, Prp19 forms tetramers via its central coiled-coil domain which are bound by Cef1.^18^ Prp19 also contains WD domains with 2 molecules of Cwc2 interacting with these WD domains in the Prp19 tetramer.^19^ The NTC is linked to the spliceosome active site through Cwc2 which interacts directly with both the U6 snRNA and the pre-mRNA.^20,21^ Recently, the *S. pombe* homolog of Cef1, called Cdc5, has been shown to bind double stranded RNA *in vitro* suggesting that Cef1/Cdc5 may also link the NTC to the active site RNAs of the spliceosome.^22^ The NTC, and NTC-associated proteins, are involved in a number of the spliceosome rearrangements and conformational changes during the splicing cycle. Associations and interactions of certain NTC proteins change following the action of certain ATPases. We now present the latest findings on the changes in the NTC that occur following ATPase action that are required for spliceosome activation, catalysis and disassembly.

## Prp2 Remodelling of the Spliceosome for the First Step of Splicing Includes NTC Protein Remodelling

During the transition of the spliceosome B complex to the B* complex, which is now competent for the first step of splicing, it is the ATPase Prp2 that makes the final rearrangement of the spliceosome.^23^ The remodelling by Prp2 involves a number of NTC proteins. Prp2 action releases the SF3a/b complex associated with the U2 snRNP.^24,25^ The removal of the SF3a/b complex creates high affinity binding sites for the NTC proteins Cwc25 and Yju2. Both Cwc25 and Yju2 are required to enable the catalytically-activated spliceosome to carry out the first step of splicing.^25,26^ The action of Prp2 requires the NTC-associated proteins Spp2 and Cwc22.^27-29^ Prp2 remodelling also involves dissociation of the NTC proteins Cwc24 and Cwc27.^7,25^ Therefore, it is clear that the action of Prp2 not only requires NTC proteins but Prp2 action results in the association and dissociation of NTC proteins with the spliceosome required for the transition through the first step of splicing.

## Prp16 Acts Through the NTC to Rearrange the Spliceosome for the Second Step of Splicing

Prp16 is an ATPase that proofreads the first step of splicing and promotes rearrangement of the spliceosome during the second step of splicing.^11,12^^,^^30-32^ During splicing a series of RNA-RNA interactions occur, and one crucial interaction is between the U2 and U6 snRNAs which base-pair to form helix I.^33^ The U2/U6 helix I is formed following the release of the U1 and U4 snRNPs, with helix I required for the first step of splicing then the second step and exon joining.^30^ It has been suggested that sequences encompassing helix I (U6 AGC triad) may form tertiary interactions with the U6 ACAGAGA box and the U6 internal stem loop (ISL) to bind a metal ion, enabling the spliceosome to have an active site resembling that found in group II introns.^34-36^ Evidence over time has pointed to a role for Prp16 in modulating a conformational change in the spliceosome involving the U2/U6 helix I.^30,31^^,^^37,38^ The U2/U6 helix I is destabilised between the 2 steps of splicing by Prp16 before helix I is reformed for the second step.^30^ As Prp16 interacts only transiently with the spliceosome, its influence on U2/U6 helix I must be applied through other spliceosome proteins. The first clue to how Prp16 could exert its action was found when deletion of the NTC protein Isy1 was shown to suppress the cold sensitive *prp16–302* allele.^38^ This was the first link between Prp16 action and the NTC complex. Recently, we have found that another NTC protein, Cwc2, stabilizes U2/U6 helix I and appears to antagonize Prp16 action.^39^ The interactions of Cwc2 with the U6 snRNA and the pre-mRNA are influenced by Prp16 mutation.^39^ The *prp16–302* allele stabilizes Cwc2 interactions with the U6 snRNA and destabilizes Cwc2 interactions with the pre-mRNA indicating that Cwc2 is one target for Prp16 action during splicing.^39^ Additionally, we have found that Cwc2 and Isy1 functionally cooperate during splicing.^39^ All together, these data point to the NTC proteins Isy1 and Cwc2, either directly or indirectly, as targets for Prp16 action in helix I remodelling during splicing.

In addition to modulating RNA-RNA and RNA-protein interactions between the 2 steps of splicing, there is evidence from both immunoprecipitation experiments and a purified yeast splicing system that Prp16 can also modulate the interactions of NTC proteins with the spliceosome prior to the second step of splicing.^8,32^ The binding of NTC proteins Cwc25 and Yju2 to the spliceosome, catalyzed by Prp2, is required to promote the first step of splicing.^25,26^ Following the first step of splicing Cwc25, and possibly Yju2, are removed to most likely allow new factors to bind to the spliceosome and promote the second step of splicing. It is the action of Prp16 that removes Cwc25, and potentially Yju2, after the first step of splicing. Using immunoprecipitation it was first shown that the action of Prp16 resulted in the release of Cwc25 and Yju2 from the spliceosome.^32^ However, recent work utilizing a purified yeast splicing system combined with mass spectrometry and dual-color fluorescence cross-correlation spectroscopy has found that Prp16 action causes a structural change in the spliceosome that reduces the binding affinity of Cwc25 allowing subsequent dissociation of Cwc25, but Prp16 action was not observed with this system to dissociate Yju2.^8^ Despite the conflicting data on Yju2 dissociation from the spliceosome, it is clear that the action of Prp16 influences the affinity of NTC proteins for the spliceosome to allow the second step of splicing.

## Prp22 Dissociates the NTC Proteins Cwc21 and Cwc22 During Spliceosome Disassembly Along with the RES Complex

Following the second step of splicing the post-splicing complex must be disassembled to release the mRNA and recycle the snRNPs. The first step of the disassembly process is carried out through the action of the ATPase Prp22.^40,41^ During splicing, the U5 snRNA interacts with the 5’ exon and 3’ exon sequences to align the 2 exons for joining during the second step of splicing.^42-47^ After the second step of splicing, the interactions of the U5 snRNA with the 2 exon sequences are disrupted by the ATPase activity of Prp22 promoting release of the mature mRNA.^48^ Recent use of the purified yeast splicing system combined with mass spectrometry to follow the spliceosome disassembly process has revealed how the protein composition of the post-splicing complex changes following Prp22 action. It was found that the NTC-associated proteins Cwc21 and Cwc22 are significantly reduced in the ILS produced by Prp22 action.^17^ In addition, the RES (REtention and Splicing) complex proteins were also found to be significantly less abundant, or absent, from the ILS following Prp22 action.^17^ The RES complex associates with the B complex along with the NTC and is required for enhancing the splicing of certain pre-mRNAs and retention of unspliced pre-mRNAs.^49-51^ Significantly, the RES complex protein Bud13/Cwc26 is an NTC protein found to associate with Cef1.^52^ Therefore, it appears that the action of Prp22 targets proteins of the NTC to induce spliceosome disassembly.

## Prp43 Disassembles the ILS to Allow Recycling of the snRNPs and the NTC for Further Rounds of Splicing

The second phase of spliceosome disassembly involves the removal of the snRNPs from the intron lariat RNA, but also dissociation of the snRNPs from each other, allowing the snRNPs to be recycled for subsequent rounds of splicing. The ATPase Prp43 is recruited for this disassembly step of the spliceosome. Prp43 associates with Ntr1 and Ntr2 (NTC-related proteins) and forms the NTR complex.^53,54^ It is Ntr1 that activates the ATPase activity of Prp43 to trigger release of the snRNPs from the intron lariat.^55^ The use of the purified yeast splicing system combined with mass spectrometry has also revealed how the protein composition of the snRNPs changes following Prp43 action to release the intron lariat and the snRNPs from each other. It has been found that the action of Prp43 completely dissociates the NTC protein Ntc20 from the snRNPs and intron-lariat.^17^ The other NTC proteins in the ILS appear to remain associated with the U2 and U5 snRNPs as well as the intron-lariat, but it is not clear how the NTC proteins are then further recycled from the released snRNPs and intron-lariat following Prp43 action.^17^ Nevertheless, it is apparent that the action of Prp43 influences NTC proteins during disassembly of the ILS.

## Brr2 is Linked to the ATPases that Remodel the NTC During Splicing

The ATPase Brr2 is an essential U5 snRNP protein involved in remodeling RNA-RNA interactions during spliceosomal activation and disassembly.^56^ Brr2 disrupts the base-pairing of the U4/U6 snRNAs to promote the release of U4, but once the catalytic steps of splicing are completed, Brr2 again disrupts the base-pairing of U2/U6 marking the start of the spliceosome disassembly process.^57-59^ Brr2 activity is regulated by the GTPase Snu114.^60^ Brr2 contains 2 helicase cassettes, with the N-terminal cassette able to hydrolyse ATP whereas the C-terminal cassette has evolved into a protein binding module.^56^ While it does not appear that Brr2 ATPase action directly influences the NTC, Brr2 is known to interact with a number of ATPases that do remodel the NTC during splicing. Brr2 has been shown to interact with Prp2 by the 2-hybrid assay but also directly by pull-down assays.^61,62^ It has been proposed that Prp2 is recruited to the spliceosome by its interaction with Brr2.^61^ Brr2 has also been shown to interact with Prp16 which may be the way in which Prp16 associates with the spliceosome.^62^ Prp43 interacts with Ntr1 and Ntr2, with Prp43 being recruited to the spliceosome through Ntr2 interaction with Brr2.^63^ Ntr2 binding to Brr2 may be prevented by Prp16 and Slu7 binding to Brr2 providing a mechanism by which Prp43 action is regulated.^64^ Overall, Brr2 appears to be a binding platform for a number of the ATPases that modulate the NTC during splicing, indirectly linking Brr2 to NTC dynamics during splicing.

## Conclusions and Future Directions

It is clear that the action of the ATPases Prp2, Prp16, Prp22 and Prp43 are related to the modulation of the NTC and NTC-associated proteins with the spliceosome during the splicing cycle. These changes in the NTC brought about by ATPase action are essential for providing the spliceosome conformations required for the first and second steps of splicing. Additionally, ATPase action is also required to modulate NTC interactions during spliceosome disassembly. Once the NTC is assimilated into the spliceosome it may not operate as a discrete complex as it appears only certain NTC proteins are modulated by ATPase action. Alternatively, it may be that the whole NTC is modulated by ATPase action but evidence is now only available for a few of the NTC proteins. In many cases it is not known whether the ATPases act directly or indirectly on the NTC proteins. In future, it will be important to determine the interaction network within the spliceosome by which the actions of the ATPases are transmitted to the NTC. There is no evidence to date for the action of the ATPases Sub2, Prp5 and Prp28 influencing the NTC as they act before the association of the NTC with the spliceosome. In humans a number of other DExD box ATPases like DDX5 (p68) and DDX17 (p72) are associated with the spliceosome.^65^ It is conceivable that the action of other ATPases may induce conformations during spliceosome assembly that allows incorporation of the NTC and NTC-associated proteins with the spliceosome. Nevertheless, the NTC appears to be a major target for ATPase remodelling of the spliceosome and the NTC is therefore intimately associated with the essential remodelling steps required for pre-mRNA splicing.
